# Optimizing immunotherapy–radiotherapy synergy in triple-negative breast cancer: mechanisms, clinical evidence, and therapeutic windows

**DOI:** 10.3389/fimmu.2026.1861526

**Published:** 2026-08-17

**Authors:** Lu Wang, Xin Bai, Fan Wang, Chenming Du, Jianming Tang

**Affiliations:** 1 The First School of Clinical Medicine, Lanzhou University, Lanzhou, Gansu, China; 2 Department of Radiation Oncology, The First Hospital of Lanzhou University, Lanzhou, Gansu, China

**Keywords:** clinical trial, immune checkpoint inhibitors, immunotherapy, radiotherapy, triple-negative breast cancer

## Abstract

Triple-negative breast cancer (TNBC) is a subtype of breast cancer associated with a poor prognosis; conventional treatments provide limited durable benefit, and resistance commonly develops. The introduction of immune checkpoint inhibitors (ICIs) has expanded treatment options for TNBC; however, ICI monotherapy has demonstrated limited efficacy, particularly in patients with advanced disease. Radiotherapy (RT) has long played a pivotal role in the treatment of early-stage and locally advanced breast cancer, as it not only exerts direct tumoricidal effects but also remodels the tumor immune microenvironment. In recent years, increasing attention has been directed toward the potential synergistic effects of ICIs and RT. This article reviews the immunological characteristics of TNBC, the current status of ICI-based therapy, and the underlying mechanisms of resistance. It also summarizes the biological rationale for combining RT with ICIs in the treatment of TNBC and, in light of recent advances in clinical research, explores the impact of different RT dose-fractionation regimens, treatment sequencing, and patient selection on therapeutic efficacy. Although current research remains in an early exploratory stage and several small studies have demonstrated the potential of this combination strategy, maximizing efficacy while controlling toxicity remains a key challenge for future clinical translation.

## 1 Introduction

**Figure 1:**
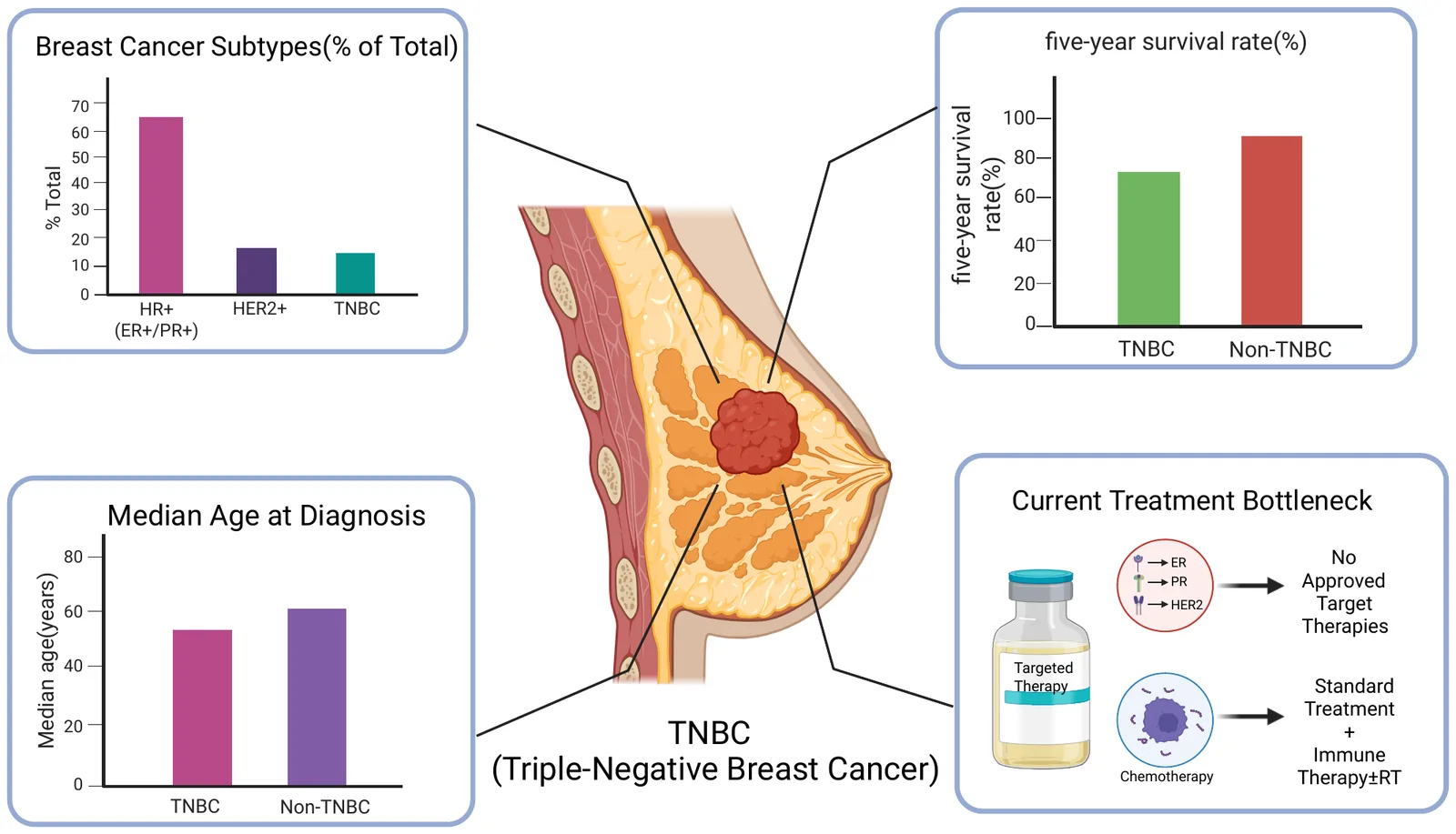
Clinical overview of TNBC. The figure summarizes the relative prevalence of TNBC among breast cancer subtypes and compares the median age at diagnosis and five-year survival between TNBC and non-TNBC. The absence of ER, PR, and HER2 limits the use of established receptor-directed therapies. Current treatment strategies rely primarily on chemotherapy, immunotherapy, RT, and selected targeted approaches according to disease stage and molecular characteristics. ER, estrogen receptor; HER2, human epidermal growth factor receptor 2; HR, hormone receptor; PR, progesterone receptor; RT, radiotherapy; TNBC, triple-negative breast cancer.

Triple-negative breast cancer (TNBC) is defined by the absence of estrogen receptor (ER) and progesterone receptor (PR) expression and human epidermal growth factor receptor 2 (HER2) overexpression or amplification, which precludes the use of established endocrine and HER2-targeted therapies (1). TNBC accounts for approximately 15%–20% of breast cancers, although its prevalence is higher among younger and non-Hispanic Black women (2, 3). It is characterized by aggressive clinical behavior, a propensity for early visceral metastasis, a high risk of early recurrence within the first few years after diagnosis, and poor outcomes after metastatic progression (3–6). Chemotherapy remains the therapeutic backbone, while PARP inhibitors, antibody–drug conjugates, and immune checkpoint inhibitors (ICIs) have broadened treatment options for selected patients (7–13) (**Figure 1**). Nevertheless, primary and acquired resistance remain common, underscoring the need for more effective combination strategies.

Radiotherapy (RT) has traditionally been used to achieve local tumor control, but its biological effects extend beyond direct cytotoxicity. RT can induce immunogenic cell death, promote tumor-antigen release and presentation, activate innate immune signaling, and facilitate effector T-cell infiltration. ICIs may complement these effects by relieving checkpoint-mediated suppression and sustaining antitumor T-cell activity, thereby providing a biological basis for combining the two modalities (14). However, RT can also trigger immunosuppressive feedback, and the balance between these opposing effects is influenced by radiation dose and fractionation, treatment sequencing, and irradiated volume.

Despite this rationale, clinical evidence for RT–immunotherapy combinations in TNBC remains limited and heterogeneous, and the patients most likely to benefit have not been clearly defined. Against this background, this review addresses a central question: What do we currently know about immunotherapy and RT in TNBC? We summarize the immunological basis for combining these modalities, examine the available preclinical and clinical evidence, and discuss safety considerations, biomarkers, and treatment variables that may guide future investigation.

## 2 Literature search and study selection

A structured literature search was conducted in PubMed/MEDLINE, Embase, and the Web of Science Core Collection from database inception through March 2026; the search was updated in July 2026 during manuscript revision. ClinicalTrials.gov was also searched to identify ongoing studies and completed trials for which full results had not been published. Search terms covered TNBC, RT, immunotherapy, immune checkpoint inhibition, dose and fractionation, treatment sequencing, the tumor immune microenvironment, immunogenic cell death, cGAS-STING signaling, Trex1, and the abscopal effect. Recent publications were prioritized, while earlier landmark studies were retained when they established key mechanistic or clinical concepts.

After duplicate records were removed, titles and abstracts were screened for relevance, and potentially eligible publications underwent full-text assessment. Clinical, translational, and preclinical studies were included when they addressed TNBC immunobiology, RT-induced immune modulation, or the efficacy, safety, dose and fractionation, and sequencing of RT–ICI combinations. Studies in other solid tumors were considered when they provided directly relevant mechanistic evidence or phase III clinical evidence. Reference lists of relevant studies and reviews were also screened. Non-oncological studies and publications outside the scope of this review were excluded. Owing to the narrative design and heterogeneity of the available evidence, the findings were synthesized qualitatively.

## 3 Immunotherapy for TNBC

### 3.1 The intrinsic immunogenicity of TNBC

Breast cancer has traditionally been regarded as an “immunologically cold” tumor, with TNBC representing a notable exception. Compared with luminal and HER2-positive subtypes, TNBC exhibits greater intrinsic immunogenicity (15). Genomic analyses have demonstrated that TNBC harbors a higher burden of somatic mutations and exhibits greater genomic instability, including homologous recombination deficiencies driven by BRCA1/2 mutations, which in turn lead to the extensive generation of neoantigens (16–18). In addition, tumor-infiltrating lymphocytes (TILs) are generally more abundant in TNBC (19, 20). Studies have shown that, among patients with TNBC who have not received neoadjuvant therapy, each 10% increase in stromal TILs is associated with an approximately 17% reduction in the risk of a distant disease-free survival event (HR = 0.83, 95% CI: 0.79–0.88), supporting the independent prognostic value of TILs (21). Programmed death-ligand 1 (PD-L1) expression provides a direct target for immunotherapy. PD-L1 is not detected in normal breast tissue but is expressed in approximately half of all breast cancers and in up to 30% of TNBC cases (22, 23). High PD-L1 expression often coexists with an immunologically activated microenvironment characterized by abundant TILs, which helps explain why PD-L1-positive patients respond better to ICIs (24). Furthermore, several pivotal phase III trials have demonstrated clinical benefits from ICI-based chemotherapy combinations in PD-L1-positive metastatic TNBC and in high-risk early-stage TNBC (25–27). TNBC is characterized by high antigenicity, substantial immune infiltration, and targetable immune evasion mechanisms. Together, these features contribute to its relative sensitivity to immunotherapy. However, this apparent immunogenicity–response paradox suggests that the presence of tumor antigens and immune infiltration does not necessarily translate into effective antitumor immunity. Instead, therapeutic outcomes may be more strongly influenced by the functional state and spatial distribution of immune cells, as well as by regulatory constraints within the tumor microenvironment.

### 3.2 Exploration of PD-1/PD-L1 inhibitors in TNBC

#### 3.2.1 Overview of PD-1/PD-L1 inhibitors

PD-1 and its principal ligand, PD-L1, are type I transmembrane proteins and key components of the immune checkpoint system. Within the tumor immune microenvironment, PD-L1, which is highly expressed on the surface of tumor cells, binds to PD-1 on T cells and delivers a potent inhibitory signal, thereby inactivating tumor-specific T cells. This interaction represents a central mechanism of immune evasion. This process is mediated by the PD-1/PD-L1 signaling pathway. Specifically, phosphorylation of tyrosine residues within the ITIM and ITSM motifs in the cytoplasmic tail of PD-1 recruits SHP-1 and SHP-2. These phosphatases then dephosphorylate signaling molecules downstream of the TCR and CD28, ultimately suppressing activation of the PI3K/Akt and Ras/MEK/Erk pathways. As a consequence, effector T-cell proliferation is impaired, the secretion of effector cytokines such as IFN-γ and TNF-α is reduced, and metabolic dysfunction ensues, culminating in a state of functional exhaustion (28, 29). In TNBC, tumor cells often exploit this inhibitory pathway in response to IFN-γ secreted by activated T cells within the tumor microenvironment. IFN-γ promotes the upregulation of PD-L1 expression on tumor cells via activation of the JAK–STAT signaling pathway, thereby establishing a self-sustaining “adaptive immune resistance” feedback loop, in which the initial immune response paradoxically reinforces the tumor’s immunosuppressive barrier (30). In response to this mechanism, immune checkpoint inhibitors targeting the PD-1/PD-L1 axis have been developed. To date, all clinically approved immune checkpoint inhibitors for breast cancer target the PD-1/PD-L1 signaling axis. Thus, monoclonal antibodies that block the interaction between PD-1 and PD-L1 can alleviate immunosuppression, restore T-cell function, and enhance antitumor immune responses (15).

Considerable uncertainty remains regarding biomarkers that predict benefit from ICIs in TNBC. Among the available candidates, PD-L1 has the greatest clinical maturity. In metastatic TNBC, PD-L1 expression assessed using the 22C3 assay and reported as a combined positive score (CPS) is used to identify patients who may benefit from pembrolizumab plus chemotherapy (25). Important strengths of PD-L1 testing include its clinical validation and widespread availability. However, its predictive accuracy remains limited. PD-L1 results depend on the antibody clone, scoring algorithm, cellular compartment assessed, and threshold used to define positivity. For example, the 22C3 CPS and SP142 immune-cell score identify overlapping but non-identical patient populations and should not be considered interchangeable (31). Pre-analytical conditions, limited biopsy material, pathologist interpretation, and spatial or temporal heterogeneity between primary and metastatic lesions may further affect PD-L1 classification (32). Treatment exposure can also alter PD-L1 expression, meaning that a single archival specimen may not accurately reflect the current immune context.

PD-L1 positivity does not guarantee response, and its predictive value differs according to disease setting. In early-stage TNBC, pembrolizumab was evaluated irrespective of PD-L1 status, and the observed benefit was not restricted to PD-L1-positive disease (33). Other biomarkers may provide complementary information, although none has been sufficiently validated to guide the selection of RT–ICI combinations. Stromal TILs can be assessed on routinely stained tissue sections and have established prognostic value in TNBC; however, lymphocyte density alone does not capture immune-cell composition, functional state, or spatial organization (21). TMB may reflect the potential for neoantigen generation, but its clinical utility is limited by the relatively low prevalence of TMB-high breast cancers and variation in sequencing platforms and cutoff values (16). Biomarker development should therefore move toward integrated and dynamic models that combine PD-L1 with immune-cell characteristics, genomic features, and circulating markers rather than relying on a single static measurement.

#### 3.2.2 Breakthroughs and paradoxes in the clinical exploration of PD-1/PD-L1 inhibitors in TNBC

On the basis of these immunological characteristics, clinical trials investigating PD-1/PD-L1-targeted ICIs in TNBC have expanded rapidly, as summarized in **Supplementary Table 1**. In the neoadjuvant setting, immunotherapy has produced clinically meaningful advances. KEYNOTE-522 (NCT03036488), a landmark phase III trial, randomized 1,174 patients with high-risk early-stage TNBC to receive pembrolizumab or placebo in combination with standard neoadjuvant chemotherapy, followed by adjuvant pembrolizumab or placebo. Among the first 602 patients included in the pCR analysis, pembrolizumab plus chemotherapy increased the pathological complete response (pCR) rate from 51.2% to 64.8% (absolute difference, 13.6%; P < 0.001). At a median follow-up of 39 months, the risk of an event affecting event-free survival was reduced by 37% (HR = 0.63, 95% CI: 0.48–0.82) (33). The IMpassion031 trial (NCT03197935), which enrolled 333 patients, similarly confirmed the value of atezolizumab in the neoadjuvant setting. The pCR rate in the atezolizumab group (n = 165) was 57.6%, significantly higher than the 41.1% observed in the chemotherapy group (absolute difference, 16.5%; 95% CI: 6–27; P = 0.0044) (34). The consistent findings of these two studies established the role of immune checkpoint inhibitors in the neoadjuvant treatment of TNBC and laid the groundwork for their subsequent application in the adjuvant setting.

However, treatment of mTNBC remains more challenging. ICI monotherapy has shown limited activity in this setting, underscoring the need for effective combination strategies (11–13). Trials evaluating ICIs in combination with chemotherapy have nevertheless yielded heterogeneous results. The IMpassion130 trial (NCT02425891; n = 902) demonstrated that atezolizumab combined with nab-paclitaxel significantly prolonged median progression-free survival (mPFS) in PD-L1-positive patients (7.5 months vs. 5.0 months; HR = 0.62, 95% CI: 0.49–0.78; P < 0.001) as well as median overall survival (mOS) (25.0 months vs. 15.5 months; HR = 0.62, 95% CI: 0.45–0.86). By contrast, the IMpassion131 trial (NCT03125902; n = 651), which used conventional paclitaxel, failed to replicate this benefit in the PD-L1-positive population (n = 292) (mPFS, 6.0 months vs. 5.7 months; HR = 0.82, 95% CI: 0.60–1.12; P = 0.20) (35, 36). This contrast suggests that the efficacy of immunotherapy may also be influenced by how the chemotherapy backbone modifies the immune microenvironment. The reasons for the discordant results remain uncertain. Differences in the chemotherapy backbone and other trial-specific factors may have contributed, but no single explanation has been established. Collectively, these studies indicate that the efficacy of combination strategies in TNBC depends strongly on treatment context, including drug selection, timing, and tumor immune status.

### 3.3 Multidimensional mechanisms of immune resistance in TNBC

**Figure 2:**
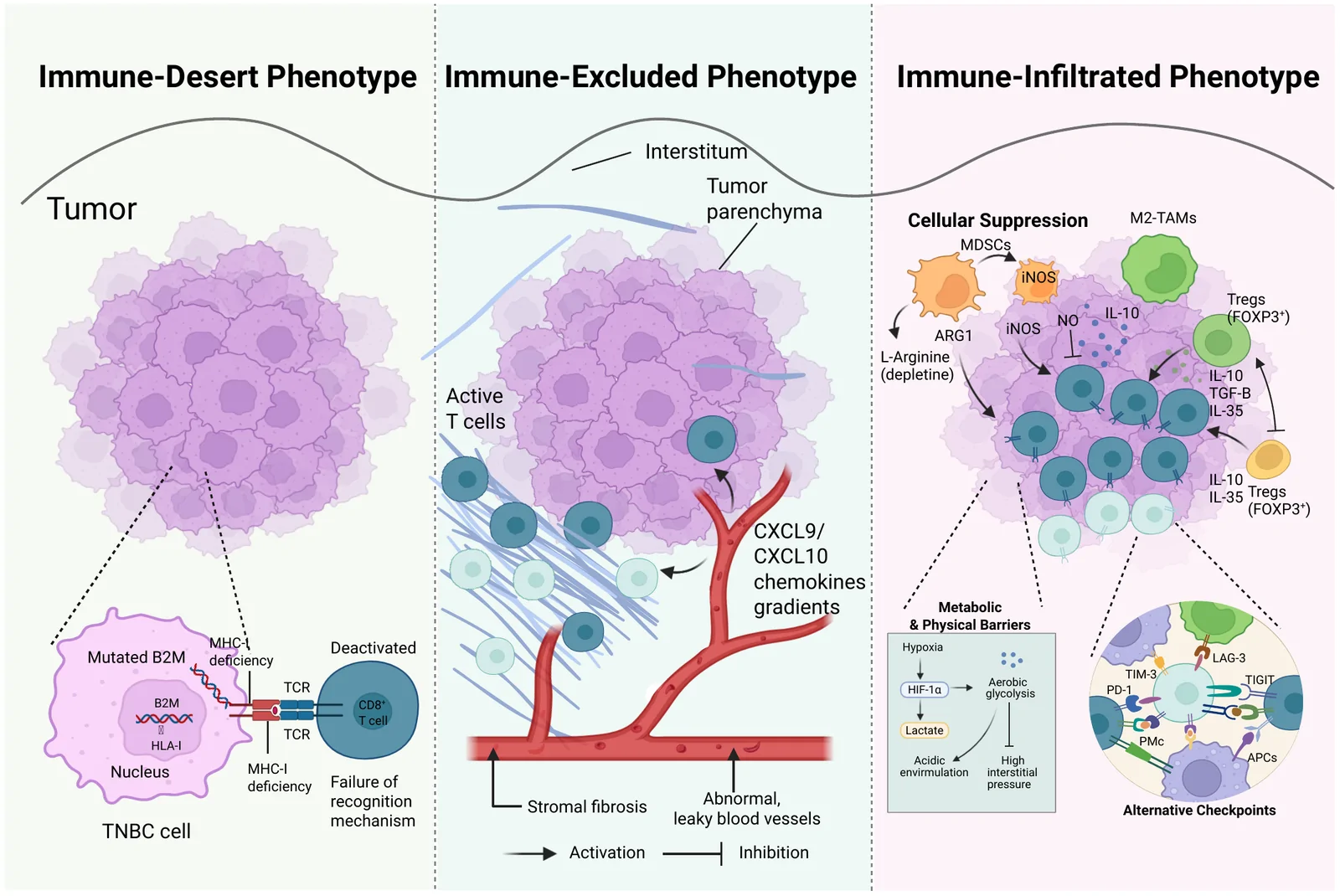
Immune resistance mechanisms in TNBC. The immune-desert phenotype is characterized by limited T-cell infiltration and impaired immune recognition. Alterations in β2M or HLA class I expression can disrupt antigen presentation through MHC class I molecules and prevent effective TCR-mediated activation of CD8^+^ T cells. In the immune-excluded phenotype, stromal fibrosis, abnormal tumor vasculature, and altered CXCL9/CXCL10 chemokine gradients restrict the migration of activated T cells from the interstitium into the tumor parenchyma. The immune-infiltrated phenotype contains abundant immune cells but remains functionally suppressed. MDSCs, M2-TAMs, and FOXP3^+^ Tregs inhibit effector T-cell activity through ARG1-mediated L-arginine depletion, iNOS-derived NO production, and the release of immunosuppressive cytokines, including IL-10, IL-35, and TGF-β. Hypoxia, HIF-1α signaling, aerobic glycolysis, lactate accumulation, extracellular acidification, and increased interstitial pressure provide additional metabolic and physical barriers. Alternative immune checkpoints, including PD-1, TIM-3, LAG-3, and TIGIT, further contribute to T-cell dysfunction and adaptive immune escape. APCs, antigen-presenting cells; ARG1, arginase 1; β2M, beta-2-microglobulin; CD8, cluster of differentiation 8; CXCL9/10, C-X-C motif chemokine ligands 9 and 10; FOXP3, forkhead box P3; HIF-1α, hypoxia-inducible factor 1α; HLA-I, human leukocyte antigen class I; IL, interleukin; iNOS, inducible nitric oxide synthase; LAG-3, lymphocyte-activation gene 3; M2-TAMs, M2-polarized tumor-associated macrophages; MDSCs, myeloid-derived suppressor cells; MHC-I, major histocompatibility complex class I; NO, nitric oxide; PD-1, programmed cell death protein 1; TCR, T-cell receptor; TGF-β, transforming growth factor beta; TIGIT, T-cell immunoreceptor with immunoglobulin and ITIM domains; TIM-3, T-cell immunoglobulin and mucin domain-containing protein 3; TNBC, triple-negative breast cancer; Tregs, regulatory T cells.

Owing to the intrinsic features and dynamic evolution of the tumor immune microenvironment, multiple mechanisms can contribute to resistance to ICI therapy in TNBC (**Figure 2**). Studies have shown that approximately 28% of TNBC cases exhibit an “immune desert” phenotype, characterized by a paucity or complete absence of T cells within both the tumor core and periphery, whereas approximately 26% display an “immune-excluded” phenotype, in which T cells are confined to the stroma and fail to penetrate the tumor parenchyma. In the former scenario, mutations in β2M or HLA class I molecules impair antigen presentation, preventing TCR recognition of antigen–MHC complexes and thereby abrogating the cytotoxic activity of T cells. This mechanism facilitates immune evasion and contributes to resistance to ICI therapy. The latter phenotype is associated with deficient expression of T-cell–recruiting chemokines such as CXCL9 and CXCL10, aberrant angiogenesis, and physical barriers imposed by dense stromal fibrosis (37–40). Even in immunologically “inflamed” TNBC, the tumor microenvironment (TME) remains enriched with diverse immunosuppressive elements. Myeloid-derived suppressor cells (MDSCs) inhibit T-cell function by depleting L-arginine through arginase-1 (ARG1) and by generating nitric oxide through inducible nitric oxide synthase (iNOS). M2-polarized tumor-associated macrophages (M2-TAMs) promote angiogenesis, extracellular-matrix remodeling, and tumor-cell migration and invasion through the secretion of IL-10, TGF-β, IL-4, and IL-13. The abundance of regulatory T cells (Tregs) is positively correlated with PD-L1 expression in TNBC. Characterized by FOXP3 expression, Tregs in TNBC suppress immune responses by consuming IL-2 and secreting immunosuppressive cytokines such as TGF-β, IL-35, and IL-10, thereby inhibiting the proliferation and function of effector T cells and promoting therapeutic resistance (41, 42). Furthermore, tumor hypoxia can activate hypoxia-inducible factor 1α (HIF-1α), while aerobic glycolysis promotes lactate accumulation within the TME, thereby establishing metabolic and physical barriers, including extracellular acidosis and elevated interstitial pressure. These factors not only directly impair T-cell function but also induce the upregulation of immune checkpoint molecules, collectively contributing to a multifaceted resistance network (43). Even in patients who initially respond to therapy, tumors may progressively evade immune surveillance through downregulation of MHC expression, activation of alternative inhibitory pathways (such as TIM-3, LAG-3, and TIGIT), or remodeling of the metabolic microenvironment (42, 44). These observations support a multidimensional approach that simultaneously targets antigen presentation, immune-cell trafficking, and metabolic suppression rather than a single resistance pathway.

## 4 Immunotherapy combined with radiotherapy for TNBC

### 4.1 Bidirectional regulatory mechanisms of radiotherapy in remodeling the immune microenvironment of TNBC

**Figure 3:**
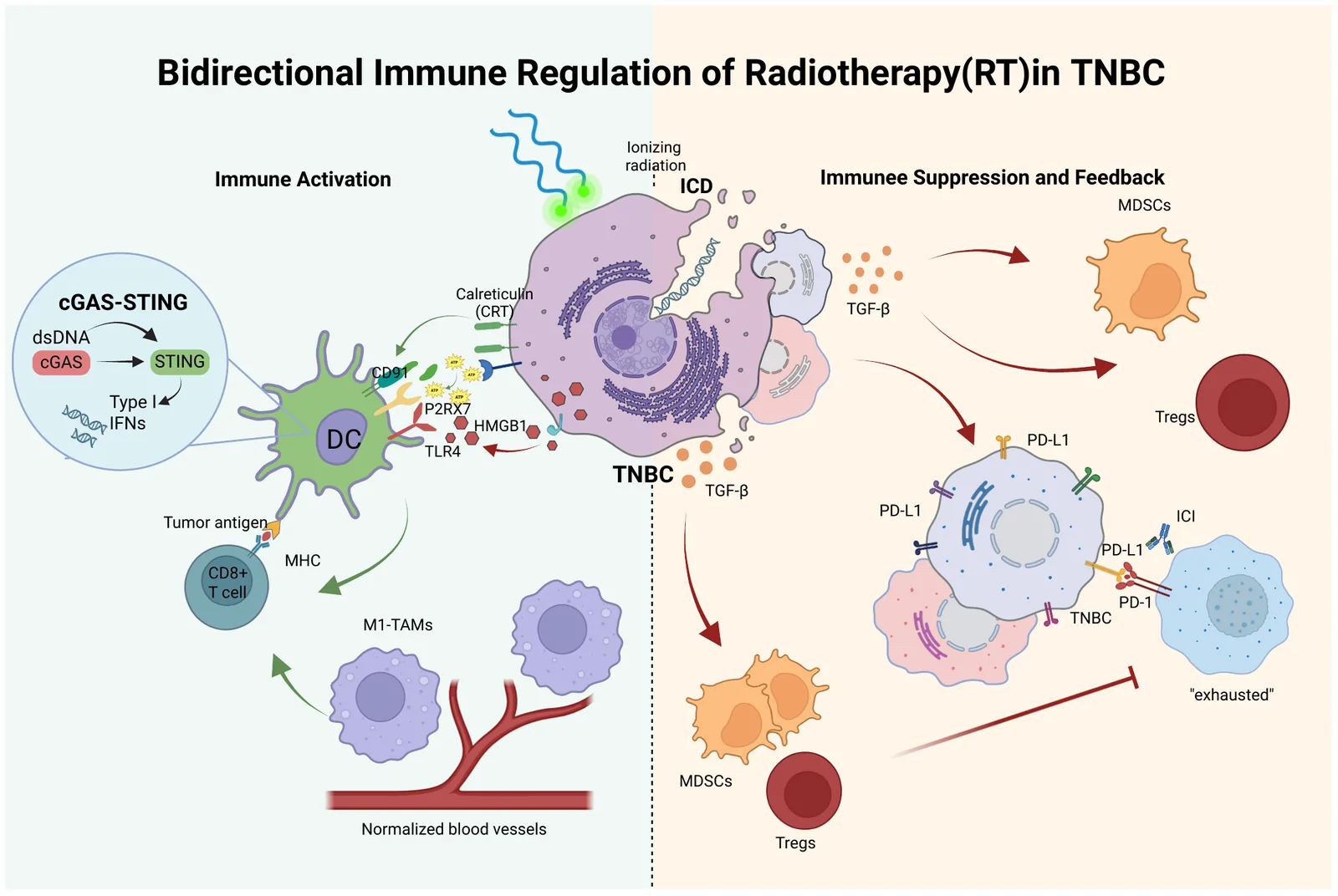
Bidirectional immune regulation of RT in TNBC. RT can promote antitumor immunity by inducing ICD and activating innate and adaptive immune responses. During ICD, surface-exposed CRT and released danger signals, including ATP and HMGB1, interact with CD91, P2RX7, and TLR4 on DCs. These interactions promote the uptake of dying tumor cells, DC maturation, and tumor-antigen presentation through MHC molecules, thereby supporting the activation of tumor-specific CD8^+^ T cells. RT-induced cytosolic dsDNA also activates the cGAS–STING pathway and stimulates type I IFN production. Vascular normalization and M1 macrophage polarization may further facilitate immune-cell trafficking and antitumor activity. In parallel, RT can induce immunosuppressive feedback through TGF-β production, the recruitment of MDSCs and Tregs, and increased PD-L1 expression on tumor cells. Engagement of PD-1 by PD-L1 contributes to T-cell dysfunction, whereas ICIs can interrupt this interaction and restore antitumor T-cell activity. The balance between these immune-stimulatory and immunosuppressive effects may influence the response to combined RT and immunotherapy. ATP, adenosine triphosphate; CD8, cluster of differentiation 8; CD91, cluster of differentiation 91; cGAS, cyclic GMP–AMP synthase; CRT, calreticulin; DCs, dendritic cells; dsDNA, double-stranded DNA; HMGB1, high-mobility group box 1; ICD, immunogenic cell death; ICIs, immune checkpoint inhibitors; IFN, interferon; M1-TAMs, M1-polarized tumor-associated macrophages; MDSCs, myeloid-derived suppressor cells; MHC, major histocompatibility complex; P2RX7, P2X purinoceptor 7; PD-1, programmed cell death protein 1; PD-L1, programmed death-ligand 1; RT, radiotherapy; STING, stimulator of interferon genes; TGF-β, transforming growth factor beta; TLR4, Toll-like receptor 4; TNBC, triple-negative breast cancer; Tregs, regulatory T cells.

The immunomodulatory effects of RT are bidirectional, as RT can induce both immune activation and immunosuppression. RT has long been a key local treatment modality for TNBC. Although RT directly induces tumor cell death through the generation of DNA double-strand breaks, its antitumor effects extend well beyond direct cytotoxicity (45). Recent studies have demonstrated that RT can also promote the release of pro-inflammatory mediators and enhance immune cell infiltration through multiple mechanisms, thereby reprogramming the TME from an immunosuppressive to an immunologically active state and providing a strong biological rationale for its combination with ICIs (**Figure 3**).

#### 4.1.1 Radiotherapy-induced immunogenic cell death and antigen release

Tumor cell death induced by RT exhibits pronounced immunogenicity, a process referred to as immunogenic cell death (ICD). Unlike conventional apoptosis, ICD does not represent a singular cell death event but rather a coordinated cascade of molecular processes. This process is accompanied by the release of damage-associated molecular patterns (DAMPs) and the secretion of immunostimulatory or chemotactic cytokines, which collectively function as endogenous “danger signals” that activate the innate immune system. Key DAMPs involved in this process include calreticulin (CRT), high-mobility group box 1 (HMGB1), and adenosine triphosphate (ATP) (46). CRT is translocated from the endoplasmic reticulum to the cell surface within 1–4 hours following irradiation, where it engages CD91 as an “eat-me” signal (47–49). Subsequently, during the early stages of apoptosis, ATP is actively secreted into the extracellular microenvironment, where it recruits dendritic cells (DCs) to the tumor site via activation of the P2RX7 receptor. During the late stages of apoptosis or secondary necrosis, HMGB1 is released from the nucleus into the extracellular space, where it binds to Toll-like receptor 4 (TLR4) on DCs, thereby promoting DC maturation and antigen presentation. Engagement of CD91, P2RX7, and TLR4 receptors on the surface of DCs facilitates the recognition and phagocytosis of dying cells, thereby enhancing antigen presentation to T cells and promoting the production of IL-1β (49, 50).

#### 4.1.2 Radiotherapy reshapes the tumor immune microenvironment

The ability of RT to reshape the tumor immune microenvironment represents a key mechanism underlying its synergy with immunotherapy. Tumor antigens released during ICD are processed and presented by DCs in draining lymph nodes, thereby priming tumor-specific CD8^+^ T cells. RT promotes effector T-cell infiltration into tumors and enhances their cytotoxic activity through multiple complementary mechanisms. RT activates the cGAS–STING signaling pathway, wherein cytosolic DNA released following irradiation is sensed by cGAS, leading to the catalytic production of cyclic GMP–AMP (cGAMP). This, in turn, activates the STING–TBK1–IRF3 signaling axis, resulting in the production of type I interferons (IFN-α/β) (51, 52). RT promotes the release of chemokines such as CXCL16 and CXCL10 from tumor cells. It also increases the expression of adhesion molecules, including E-selectin and ICAM-1, on endothelial cells. In addition, RT upregulates MHC class I, Fas, ICAM-1, and NKG2D ligands. Together, these changes enhance immune recognition and response (53–59). RT can further remodel the tumor vasculature by promoting vascular normalization via downregulation of vascular endothelial growth factor (VEGF) and angiopoietin-2 (Ang-2), thereby improving perfusion and reducing interstitial fluid pressure, ultimately alleviating physical barriers to immune cell infiltration (60). Furthermore, RT promotes the polarization of TAMs, shifting them from the M2 to the M1 phenotype. M1 macrophages secrete pro-inflammatory cytokines, including TNF-α and IL-12, thereby enhancing antitumor immune responses (61). Collectively, these mechanisms constitute the RT-mediated “immune sensitization” effect.

#### 4.1.3 Influence of radiation dose and fractionation on immunogenicity

Importantly, the immune-activating effects of RT are highly dependent on dose and fractionation. Arnold et al. reported that fractionated RT at 4 Gy × 2 fractions promoted the polarization of TAMs from the M2 to the M1 phenotype, whereas this effect was absent in the high-dose group (61). These findings further highlight the dose-dependent impact of RT on the tumor immune microenvironment. Current preclinical studies suggest that moderately hypofractionated RT, approximately 8–12 Gy per fraction, may favor immune activation in selected experimental settings; however, prospective clinical validation in TNBC remains limited. By contrast, doses above a tumor-dependent threshold of approximately 12–18 Gy can induce the DNA exonuclease Trex1, leading to the degradation of cytosolic DNA and attenuation of cGAS–STING-mediated type I interferon signaling, thereby diminishing immunogenicity (62). This dual effect indicates a nonlinear, context-dependent relationship between radiation dose and RT-induced immune activation.

In a seminal 2009 study, Dewan et al. evaluated three RT regimens—20 Gy × 1, 8 Gy × 3, and 6 Gy × 5—in combination with the anti-CTLA-4 antibody 9H10 in a murine TSA breast cancer model. The 8 Gy × 3 and 6 Gy × 5 regimens induced abscopal effects, achieving complete regression in 20–40% of distant tumors, whereas a single 20 Gy dose failed to produce such responses (63). Consistent with these findings, Schaue et al. found that 7.5 Gy per fraction provided a favorable balance between tumor control, antitumor immunity, and Treg accumulation (64). Grapin et al. subsequently reported that 8 Gy × 3 showed the greatest therapeutic activity among the tested regimens when combined with PD-L1 and TIGIT blockade (65). Collectively, these findings support moderately hypofractionated RT, particularly regimens around 8 Gy per fraction, as a rational candidate for immune modulation. However, the most appropriate dose and fractionation are likely to vary according to tumor type, irradiated volume, immune context, and the accompanying systemic treatment.

#### 4.1.4 Radiotherapy-induced distant effects and the theoretical basis for combined therapy

Following local RT, regression of non-irradiated distant lesions can occur. This phenomenon, in which tumor control extends beyond the irradiated field, is referred to as the “abscopal effect,” a concept first described by R. H. Mole in 1953 (66). Although uncommon with RT alone, the abscopal effect is enhanced when RT is combined with ICIs (63, 67). This observation likely reflects the dynamic interplay between RT-induced immunogenic signals and concurrent immunosuppressive effects. RT increases TGF-β levels, promotes the recruitment of MDSCs, and enhances the accumulation of Tregs. It also upregulates PD-L1 expression in tumor cells. Together, these changes contribute to a strongly immunosuppressive microenvironment (68–70). Although RT activates antitumor immune responses, it simultaneously initiates feedback mechanisms that facilitate tumor immune escape. Administering immunotherapy during this window can block the PD-1/PD-L1 axis. This approach counteracts RT-induced compensatory immunosuppression and enables activated effector T cells (Teffs) to maintain their cytotoxic function.

### 4.2 Clinical research progress of ICIs combined with radiotherapy in the treatment of TNBC

Building on these biological mechanisms, clinical investigation of RT–ICI combinations in TNBC is expanding, although the field remains at an early stage. The available and ongoing clinical studies are summarized in **Supplementary Table 2**. Compared with other malignancies, such as non-small cell lung cancer and melanoma, prospective randomized Phase III data in TNBC remain scarce. Most of the available clinical evidence is derived from small Phase II studies (71–76). These studies have predominantly enrolled patients with metastatic TNBC and have focused on the safety and preliminary efficacy of combination approaches.

#### 4.2.1 Clinical research progress of metastatic/recurrent TNBC

In the setting of mTNBC, the TONIC trial (NCT02499367) was a multi-arm, randomized phase II trial that evaluated nivolumab administered sequentially after various induction therapies. In the RT arm (n = 12), patients received high-dose fractionated RT (8 Gy × 3 fractions), and the final ORR was approximately 8% (95% CI: 0.2%–38.5%). This was lower than the 35% observed in the doxorubicin induction group (n = 17; 95% CI: 14.2%–61.7%) and the 23% reported in the cisplatin induction group (n = 13; 95% CI: 5.0%–53.8%). The TONIC trial demonstrated that RT induction resulted in a relatively low response rate compared with other induction strategies, suggesting that radiotherapy alone may be insufficient to effectively prime systemic antitumor immunity in mTNBC. One possible explanation is the limited irradiation field, as single-lesion RT may be insufficient to overcome systemic immunosuppression, particularly that mediated by MDSCs. These findings indicate that the efficacy of RT–ICI combinations depends not only on immune activation but also on the ability to modulate systemic immunosuppression (71). In contrast, Ho et al. conducted a phase II single-arm study (NCT02730130) to evaluate the efficacy and safety of pembrolizumab combined with SBRT (3,000 cGy delivered in 5 fractions) in metastatic TNBC, with pembrolizumab administered within 3 days of the first RT session. The combination of pembrolizumab and RT was well tolerated and showed preliminary antitumor activity. The overall ORR was 17.6% (3 of 17 patients; 95% CI: 4.7%–44.2%). Among the 9 evaluable patients, 3 (33%) achieved complete responses (CR) in distant lesions outside the irradiated target area, and the longest duration of remission exceeded 108 weeks. Responses observed in non-irradiated lesions provide preliminary evidence that appropriately integrated RT may contribute to systemic antitumor immunity. The favorable outcomes may partly reflect the synchronous administration of RT and ICIs, which could improve coordination between antigen release and T-cell activation. These findings highlight the importance of treatment timing as a critical determinant of therapeutic efficacy in RT–ICI combinations (72). The NCT06735131 trial is a prospective, multicenter study designed to explore the optimal integration of RT and immunotherapy in mTNBC and to compare the effects of different RT fractionation regimens on immune activation. Recruitment is currently ongoing. The trial includes five different RT regimens: 5 Gy × 5 fractions once daily; 8 Gy × 5 fractions once daily; 8 Gy × 3 fractions every other day; 10 Gy × 3 fractions every other day; and 0.5 Gy twice daily for 2 days, repeated for 4 cycles (total dose, 8 Gy), all in combination with toripalimab and chemotherapy. The primary endpoint is the 6-month PFS rate. This study is expected to provide prospective evidence to guide the selection of RT fractionation regimens.

Several studies aimed at optimizing RT–immunotherapy combination strategies are currently underway. To address myeloid-mediated immunosuppression, the ASTRAEA trial (NCT07015853) is evaluating a combination strategy incorporating an MDSC-targeting agent. This is the first trial to combine radiotherapy (SBRT, 24 Gy in 3 fractions) with the CSF1R inhibitor axatilimab and the PD-1 inhibitor pembrolizumab. This strategy is intended to activate antitumor immunity through RT while mitigating myeloid-mediated suppression with axatilimab. Beyond targeting MDSCs, strategies employing oncolytic viruses to further enhance RT-induced immunogenicity are also under investigation. The STOMP trial (NCT03004183) is a Phase II single-arm study evaluating a regimen of radiotherapy (SBRT, 30 Gy in 5 fractions) combined with an oncolytic virus (ADV/HSV-tk), valacyclovir, and pembrolizumab in patients with mTNBC. The primary endpoint is objective response rate (ORR). This study employed a sequential design. SBRT and oncolytic virus therapy were administered concurrently from days 2 to 16 to prime immune activation. Pembrolizumab was initiated on day 17 and continued thereafter to sustain the response. The clinical benefit rate was 21.43% (6/28). including 2 complete responses (7.1%), 1 partial response (3.6%), and 3 cases of durable stable disease (≥ 24 weeks, 10.7%) (73). These preliminary findings support further evaluation of immune-stimulatory strategies designed to enhance RT-induced immunogenicity. Oncolytic viruses directly lyse tumor cells, leading to antigen release. This effect synergizes with RT-induced ICD to amplify the “*in situ* vaccine” effect, thereby promoting DC maturation and T-cell infiltration (77). These findings support the concept that combinatorial strategies targeting multiple steps of the cancer–immunity cycle may be required to overcome immune resistance in mTNBC. The NADiR trial (NCT04837209) is a Phase II study investigating the combination of radiotherapy with the PD-1 inhibitor dostarlimab and the PARP inhibitor niraparib in patients with mTNBC. By impairing DNA damage repair in BRCA-wild-type TNBC, PARP inhibitors may enhance RT-induced immunogenicity and help overcome primary and acquired immune resistance. Importantly, this study enrolls patients who are PD-L1-negative or have previously failed ICI therapy, representing a particularly challenging population for immunotherapy. The trial therefore evaluates PARP inhibition beyond BRCA-mutated disease in a broader population with immune-resistant TNBC. RT is initiated concurrently with the PARP inhibitor on day 1 of cycle 1, based on the premise that increasing cumulative DNA damage may enhance RT-induced immunogenicity, amplify cGAS–STING signaling, and improve responsiveness to immunotherapy. This design reflects a shift from dual- to multimodality combinations aimed at enhancing immunogenicity while relieving immunosuppression. For patients with mTNBC and brain metastases—a population with an extremely poor prognosis—the NCT06238921 trial is a Phase I/II single-arm study evaluating the safety and efficacy of stereotactic radiosurgery (SRS) in combination with the antibody–drug conjugate (ADC) sacituzumab govitecan and the PD-1 inhibitor zimberelimab. The primary endpoint in Phase I is neurological toxicity. In Phase II, the primary endpoint is progression-free survival (PFS). As a Trop-2-targeted ADC, sacituzumab govitecan has demonstrated the ability to penetrate the blood–brain barrier (BBB) to a certain extent, enabling delivery to intracranial tumors (74). Radiotherapy not only provides local control of intracranial lesions but also increases BBB permeability, thereby enhancing intracranial delivery of ADCs. In parallel, RT induces immunogenic cell death, while PD-1 blockade can reverse T-cell exhaustion and restore antitumor immune activity (78). This study represents the first clinical attempt to integrate ADCs, RT, and immunotherapy for TNBC brain metastases, an area of critical unmet need. If successful, the trial may inform a new treatment approach for this population and broaden the application of RT–immunotherapy combinations to site-specific metastatic disease.

#### 4.2.2 Clinical research progress of early TNBC

In the neoadjuvant and adjuvant management of early-stage TNBC, the establishment of the KEYNOTE-522 regimen has prompted investigators to explore the integration of RT into treatment strategies. In the Phase Ib/II PEARL trial (NCT03366844), 66 patients with HER2-negative breast cancer, including 54 with TNBC, received pembrolizumab together with local RT to the primary tumor site (8 Gy × 3 fractions, once daily) before standard neoadjuvant chemotherapy. The regimen was well tolerated and achieved a pathological complete response (pCR) rate of 59.2% (32/54) in the TNBC cohort. Although the addition of RT significantly upregulated tumor PD-L1 expression, with the median CPS increasing from 8.65 to 26.15 (95% CI: –46.61 to –4.04; P = 0.021), it was also associated with a significant reduction in the proportion of TILs, from 28.9% to 17.1% (95% CI: 2.46–21.09; P = 0.014) (75). The decrease in TILs observed after RT in early-stage TNBC may reflect transient immunosuppression. Alternatively, it may indicate redistribution of TILs from the tumor parenchyma to lymph nodes or peripheral blood rather than true functional loss. Moreover, stromal TIL levels may increase after surgery and have been associated with improved clinical outcomes. Accordingly, although RT may induce short-term immune cell depletion or redistribution, its overall impact may be more appropriately characterized as a systemic immune-enhancing effect. Biomarkers measured at a single time point may therefore not fully capture the dynamic immune changes induced by RT. Despite its limited sample size, the study provides direct prospective evidence of the dynamic interplay between RT and immune regulation in humans. This exploratory study provides a biological and clinical rationale for subsequent randomized phase III trials evaluating RT–immunotherapy combinations in early-stage TNBC.

Building on the PEARL trial, the NCT05132790 study further investigated treatment sequencing and dose fractionation strategies. This pilot study assessed the efficacy and safety of neoadjuvant stereotactic body RT (SBRT) in combination with the PD-L1 inhibitor adebrelimab and standard chemotherapy in patients with early-stage TNBC. In this protocol, SBRT (8 Gy × 3 fractions, administered every other day) was delivered at the initiation of the second cycle of adebrelimab, followed by six cycles of adebrelimab in combination with nab-paclitaxel and carboplatin. The therapeutic activity observed was encouraging. A pCR rate of 90% (9/10; 95% CI: 55.5–99.8%) was achieved, with both the residual cancer burden (RCB 0–I) rate and overall response rate (ORR) reaching 100% (95% CI: 67.9–100.0%). Among the 13 patients included in the safety analysis, any-grade AEs occurred in 92.3%, whereas grade 3 or higher treatment-related AEs occurred in 53.8%. One patient experienced a treatment-related serious AE, while no radiation-related dermatitis or treatment-related death was reported. Collectively, these findings provide early clinical support for further evaluation of SBRT administered after the initiation of immunotherapy (76). However, the small sample size and multimodal treatment design preclude conclusions regarding the independent contribution of RT or the optimal dose and treatment sequence. Larger randomized studies are required to determine whether the addition of RT provides further benefit beyond immunotherapy and chemotherapy alone.

To validate these observations, larger confirmatory studies are currently in progress. The NCT06627712 trial is an ongoing multicenter Phase III study designed to evaluate the clinical benefit of SBRT (24 Gy in 3 fractions, administered every other day) in combination with a PD-1 inhibitor and chemotherapy in the neoadjuvant setting for early-stage TNBC. In this trial, 318 patients with cT1cN1-2M0 or cT2N0-2M0 disease are being randomized to receive either SBRT plus immunotherapy and chemotherapy or immunotherapy combined with chemotherapy alone, with pCR as the primary endpoint. A distinctive feature of this study is the integration of SBRT into the established KEYNOTE-522 treatment framework. This design enables a direct evaluation of whether the addition of RT to standard immunotherapy and chemotherapy can further improve pCR rates. The results may clarify whether adding RT should be incorporated into neoadjuvant treatment for early-stage TNBC.

Overall, the reported efficacy of RT–ICI combinations in TNBC has been heterogeneous and appears to depend on radiation dose, fractionation, treatment sequencing, and patient selection. Future trials should therefore incorporate patient stratification based on immune phenotype and tumor burden to identify the subgroups most likely to benefit from RT–ICI combinations. However, the lack of standardized treatment protocols and the heterogeneity of clinical trial designs remain major challenges for clinical application. Future studies should focus on defining context-specific dose, fractionation, and sequencing strategies and on integrating RT with approaches that address systemic immune suppression.

### 4.3 Safety and toxicity management of combination therapy

Although the combination of immunotherapy and RT offers a promising new therapeutic strategy for TNBC, it may also introduce additional toxicities. From an overall safety standpoint, a pooled analysis of 68 prospective ICI trials in the U.S. Food and Drug Administration database included 16,835 patients, of whom 1,733 had received RT within 90 days before ICI initiation. Overall adverse-event rates were generally similar between patients with and without recent RT, and no meaningful increase in grade 3–4 adverse events was observed (79). These findings provide reassurance regarding the sequential administration of RT and ICIs. In patients with TNBC, the PEARL trial demonstrated that neoadjuvant RT at 8 Gy × 3 fractions combined with pembrolizumab was well tolerated. The incidence of grade 3 or higher toxicity during the overall treatment period was 40.9%, and no delay in subsequent chemotherapy was observed. Importantly, this cumulative toxicity was primarily attributable to the subsequent phase of standard chemotherapy (75). By contrast, the BREASTIMMUNE-03 trial (NCT03818685; n = 95) evaluated the addition of dual immunotherapy with nivolumab and ipilimumab (n = 45) to postoperative RT in patients with TNBC and residual disease after neoadjuvant chemotherapy and surgery, with capecitabine serving as the comparator (n = 50). Recently reported results showed no significant improvement in DFS. Moreover, the study was terminated early because of a high incidence of myocarditis in the immunotherapy group (5/45, 11%) (80). This negative finding serves as an important cautionary signal, indicating that excessive intensification of immunotherapy in the setting of minimal residual disease does not necessarily translate into survival benefit. These results underscore the need to balance immune activation against toxicity rather than pursue maximal immune stimulation. Beyond clinical trials, real-world evidence provides important complementary insights into the safety of combined immunotherapy and RT in the adjuvant setting. The INT 54/24 study prospectively collected data from 10 patients with early-stage TNBC who received adjuvant pembrolizumab concurrently with RT after treatment according to the KEYNOTE-522 regimen. The incidence of serious toxicity was 20% (2/10), including one case of grade 4 myositis and one case of grade 3 electrolyte disturbance, whereas the remaining patients demonstrated good overall tolerability. These preliminary real-world data support the feasibility of concurrent adjuvant pembrolizumab and RT after the KEYNOTE-522 regimen, although the cohort was small (81).

These observations highlight that the safety of RT–ICI combinations is highly context-dependent and influenced by treatment intensity, disease burden, and patient-specific factors. Future strategies should aim to personalize combination regimens by optimizing treatment intensity and avoiding unnecessary immune overactivation, particularly in early-stage or minimal disease settings. Overall, current evidence suggests that the toxicity profile of RT–ICI combinations is not purely additive but reflects a complex interplay between immune activation and tissue-specific responses. A deeper understanding of these mechanisms will be essential for developing safer and more effective combination strategies.

## 5 Discussion

RT combined with immunotherapy represents a promising but complex therapeutic strategy for TNBC. However, current clinical evidence remains insufficient to define an optimal treatment approach. Most available studies are small, single-arm trials with substantial variation in disease stage, irradiated targets, RT dose and fractionation, systemic therapy, treatment sequencing, and clinical endpoints. The reported antitumor activity should therefore be interpreted cautiously, because the local effects of RT, concomitant chemotherapy, and patient selection may all contribute to the observed outcomes. Although moderately hypofractionated regimens, particularly those delivering approximately 8 Gy per fraction, have shown favorable immune effects in several preclinical models, these findings do not define a universal immunological window. The optimal dose and fractionation are likely to vary with tumor type, irradiated volume, immune context, and concomitant systemic therapy; their clinical relevance in TNBC remains to be established.

Clinical experience in other malignancies provides useful context, although disease settings and treatment schedules differ. In unresectable stage III non-small cell lung cancer, the 713-patient PACIFIC trial established durvalumab after chemoradiotherapy as an effective consolidation strategy and demonstrated durable survival benefits (82). More direct evidence has emerged from high-risk locally advanced cervical cancer. In the 1,060-patient KEYNOTE-A18 trial, pembrolizumab was administered concurrently with chemoradiotherapy and subsequently continued as maintenance therapy, resulting in significant improvements in progression-free and overall survival (83). However, similar strategies have not been successful in all tumor types. In the 697-patient JAVELIN Head and Neck 100 trial, the addition of concurrent and maintenance avelumab to chemoradiotherapy failed to improve progression-free survival (84). Collectively, these findings indicate that the clinical benefit of integrating RT with immunotherapy depends on tumor type, disease setting, treatment sequence, and the accompanying systemic regimen. These cross-tumor findings also caution against attributing favorable outcomes directly to an abscopal effect.

Beyond refining RT dose, fractionation, and treatment sequencing, future studies should evaluate mechanism-based combinations that address specific barriers to antitumor immunity. STING agonists represent one such approach. Pharmacological STING activation could reinforce RT-induced type I interferon signaling and dendritic-cell priming when endogenous cGAS–STING activity is insufficient. In an early-phase study of 34 patients with ICI-refractory solid tumors, including nine with TNBC, hypofractionated RT followed by pembrolizumab and the systemic STING agonist dazostinag was associated with pharmacodynamic activation of STING- and IFN-γ-related pathways (85). However, the small TNBC subgroup and limited number of responses preclude conclusions regarding clinical efficacy in this disease. Oncolytic and other intratumoral virus-based therapies offer a complementary strategy. By promoting tumor-cell lysis, antigen release, and local inflammation, these agents may strengthen the *in situ* vaccination effect of RT. The STOMP trial provided early clinical evidence for combining virus-based therapy, SBRT, and pembrolizumab in mTNBC, although its small, single-arm design did not allow the contribution of each treatment component to be determined (73).

Other emerging strategies seek to broaden or redirect T-cell activity. Bispecific antibodies can simultaneously inhibit two immune checkpoints or recruit T cells directly to tumor-associated antigens. Early clinical activity has been reported in a phase II study of the PD-L1/CTLA-4 bispecific antibody KN046 plus nab-paclitaxel in 27 patients with mTNBC, 25 of whom were evaluable for response, whereas TROP2×CD3 constructs have shown antitumor activity mainly in preclinical TNBC models (86, 87). Their integration with RT remains investigational and requires careful evaluation of cytokine-mediated and off-tumor toxicities. Targeting LAG-3, TIGIT, or TIM-3 may also help overcome adaptive resistance to PD-1/PD-L1 blockade, particularly when these inhibitory pathways are upregulated during persistent immune stimulation (65, 88, 89). Nevertheless, evidence in TNBC remains preliminary, and combining RT with multiple checkpoint inhibitors may increase immune-related toxicity. Future trials should use longitudinal biomarkers to identify the dominant resistance pathway and guide the selection of rational combinations, including approaches directed against MDSCs and TAMs. The objective should be coordinated immune modulation with acceptable toxicity rather than indiscriminate treatment intensification.

## 6 Limitations of the review

This review has several limitations. Although a structured search strategy was used to identify relevant studies, the review was narrative in design and did not include a formal risk-of-bias assessment or quantitative evidence synthesis. The selection and interpretation of evidence may therefore be subject to bias. More importantly, clinical evidence specifically evaluating RT–ICI combinations in TNBC remains limited. Most available studies are small, single-arm, early-phase trials, with substantial variation in disease stage, irradiated targets, RT dose and fractionation, checkpoint inhibitors, concomitant systemic therapy, treatment sequence, and clinical endpoints. These differences limit direct comparisons across studies and preclude definitive conclusions regarding the optimal treatment strategy. In addition, several ongoing trials are represented only by registry information or preliminary reports, and their final results may differ from the evidence available at the time of this review.

Many mechanistic conclusions are also derived primarily from preclinical models. In particular, the immunogenic effects reported with moderately hypofractionated regimens, including 8 Gy × 3, remain model-dependent. An optimal dose or fractionation schedule has not been clinically established in TNBC, and the observed immune effects may vary according to tumor genotype, irradiated volume, anatomical site, and concomitant systemic therapy. Evidence from other malignancies provides useful clinical context, but its direct application to TNBC remains limited because of differences in tumor biology, treatment intent, radiation fields, and immunotherapy schedules. These limitations highlight the need for prospective randomized studies with standardized RT parameters, clinically meaningful endpoints, and integrated biomarker analyses.

## 7 Conclusion

RT and immunotherapy are complementary in TNBC because they engage different stages of antitumor immunity. By promoting tumor-antigen release, innate immune sensing, and T-cell trafficking, RT can create conditions that favor immune activation; ICIs may then sustain these responses by relieving checkpoint-mediated suppression. Yet this biological rationale has not translated into consistent clinical benefit, underscoring the need for biomarker-guided patient selection and treatment design. Given the spatial and temporal heterogeneity of the TNBC immune microenvironment, PD-L1 alone is unlikely to provide sufficient predictive information. Integrated assessment of PD-L1, TIL characteristics, immune phenotype, genomic features, and circulating markers may better identify patients most likely to benefit and inform the selection of RT dose, fractionation, and treatment sequence.

## Glossary

ADCs: antibody–drug conjugates
AEs: adverse events
ATP: adenosine triphosphate
BBB: blood–brain barrier
BRCA1/2: breast cancer susceptibility genes 1 and 2
cGAMP: cyclic GMP–AMP
cGAS: cyclic GMP–AMP synthase
CI: confidence interval
CPS: combined positive score
CR: complete response
CRT: calreticulin
CTLA-4: cytotoxic T-lymphocyte-associated protein 4
DAMPs: damage-associated molecular patterns
DCs: dendritic cells
DDFS: distant disease-free survival
DFS: disease-free survival
EFS: event-free survival
ER: estrogen receptor
FDA: U.S. Food and Drug Administration
HER2: human epidermal growth factor receptor 2
HIF-1α: hypoxia-inducible factor 1 alpha
HMGB1: high-mobility group box 1
HR: hazard ratio
ICD: immunogenic cell death
ICIs: immune checkpoint inhibitors
IFN: interferon
IL: interleukin
IRF3: interferon regulatory factor 3
ITIM: immunoreceptor tyrosine-based inhibitory motif
ITSM: immunoreceptor tyrosine-based switch motif
JAK: Janus kinase
LAG-3: lymphocyte-activation gene 3
MDSCs: myeloid-derived suppressor cells
MHC: major histocompatibility complex
mOS: median overall survival
mPFS: median progression-free survival
mTNBC: metastatic triple-negative breast cancer
ORR: objective response rate
OS: overall survival
PARP: Poly (ADP-ribose) polymerase
pCR: pathological complete response
PD-1: programmed cell death protein 1
PD-L1: programmed death-ligand 1
PFS: progression-free survival
PI3K: phosphoinositide 3-kinase
PR: progesterone receptor
RCB: residual cancer burden
RT: radiotherapy
SBRT: stereotactic body radiotherapy
SD: stable disease
SHP-1/2: Src homology 2 domain-containing phosphatases 1 and 2
SRS: stereotactic radiosurgery
STAT: signal transducer and activator of transcription
STING: stimulator of interferon genes
TAMs: tumor-associated macrophages
TBK1: TANK-binding kinase 1
TCR: T-cell receptor
Teffs: effector T cells
TGF-β: transforming growth factor beta
TIGIT: T-cell immunoreceptor with immunoglobulin and ITIM domains
TILs: tumor-infiltrating lymphocytes
TIM-3: T-cell immunoglobulin and mucin domain-containing protein 3
TMB: tumor mutational burden
TME: tumor microenvironment
TNBC: triple-negative breast cancer
TNF-α: tumor necrosis factor alpha
Tregs: regulatory T cells
Trex1: three-prime repair exonuclease 1
Trop-2: trophoblast cell-surface antigen 2
VEGF: vascular endothelial growth factor
