# Supplementary material for: Optimizing immunotherapy–radiotherapy synergy in triple-negative breast cancer: mechanisms, clinical evidence, and therapeutic windows
Source: Front Immunol. 2026 Aug 17;17:1861526. doi: 10.3389/fimmu.2026.1861526 (PMC13527378; doi:10.3389/fimmu.2026.1861526)
Supplement: Supplementary file 1 [file Table1.docx]

Supplementary Material

| Trial | Phase | Setting | PD1/PD-L1 inhibitor | Drug administration regimen | Sample size | Result | Status |
| --- | --- | --- | --- | --- | --- | --- | --- |
| KEYNOTE-522 (NCT03036488) | Ⅲ | Early-stage TNBC | Pembrolizumab(200 mg, IV, on Day 1 of each cycle) | Pembrolizumab / Placebo + Paclitaxel + Carboplatin → Pembrolizumab / Placebo + Doxorubicin / Epirubicin + Cyclophosphamide → Pembrolizumab / Placebo | 1174 | pCR:64.8% vs 51.2%  EFS：HR=0.63 | complete |
| IMpassion031 (NCT03197935) | Ⅲ | Early-stage TNBC | Atezolizumab(840 mg, IV, 1200 mg, IV, post-surgery) | Atezolizumab / Placebo + nab-Paclitaxel → Atezolizumab / Placebo + Doxorubicin + Cyclophosphamide → Atezolizumab | 333 | ITT: 58% vs 41%; PD-L1+: 69% vs 49% | complete |
| IMpassion130 (NCT02425891) | Ⅲ | Locally advanced or Metastatic TNBC | Atezolizumab(840 mg, IV, on Day 1, 15 of each cycle) | Atezolizumab / Placebo + nab-Paclitaxel | 902 | ITT: mPFS 7.2 vs 5.5 mo; mOS 21.3 vs 17.6 mo  PD-L1+: mPFS 7.5 vs 5.0 mo; mOS 25.0 vs 15.5 mo | complete |
| IMpassion131 (NCT03125902) | Ⅲ | Locally advanced or Metastatic TNBC | Atezolizumab(840 mg, IV, on Day 1, 15 of each cycle) | Atezolizumab / Placebo + Paclitaxel | 651 | PD-L1+: mPFS 6.0 vs 5.7 mo | complete |
| ASCENT-04 (NCT05382286) | Ⅲ | Locally advanced or Metastatic TNBC | Pembrolizumab(200 mg, IV, on Day 1 of each cycle ) | Sacituzumab govitecan / Chemotherapy + Pembrolizumab | 443 | mPFS: 11.2 vs 7.8 mo | Active, not recruiting |
| KEYNOTE-355 (NCT02819518) | Ⅲ | Locally advanced or metastatic TNBC | Pembrolizumab(200 mg, IV, on Day 1 of each cycle ) | Pembrolizumab / Placebo + nab-Paclitaxel / Paclitaxel / Gemcitabine / Carboplatin | 847 | CPS≥10: mPFS 9.7 vs 5.6 mo ; CPS≥1: mPFS 7.5 vs 5.6 mo ; ITT: mPFS 7.6 vs 5.6 mo | complete |
| KEYNOTE-119 (NCT02555657) | Ⅲ | Metastatic TNBC | Pembrolizumab(200 mg, IV) | Pembrolizumab / Chemotherapy | 622 | CPS≥10: mOS 12.7 vs 11.6 mo; CPS≥1: mOS 10.7 vs 10.2mo; overall:mOS 9.9 vs 10.8mo | complete |
| GeparNuevo (NCT02685059) | Ⅱ | Early-stage TNBC | Durvalumab(1.5g, IV) | Durvalumab / Placebo → Durvalumab / Placebo + nab-Paclitaxel → Durvalumab + EC | 174 | pCR: 53.4% vs 44.2% | complete |
| NCT02620280 | Ⅲ | Early high-risk and locally advanced TNBC | Atezolizumab(1200 mg, IV, on Day 1 of each cycle ) | Carboplatin + Abraxane ± Atezolizumab → AC / EC / FEC | 280 | pCR: 48.6% vs 44.4% | complete |
| KEYNOTE-086 (NCT02447003) | Ⅱ | Metastatic TNBC | Pembrolizumab(200 mg, IV, on Day 1 of each cycle ) | Pembrolizumab | 254 | ORR: 5.3%; PD-L1+ ORR: 5.7% | complete |
| A-BRAVE (NCT02926196) | Ⅲ | High-risk TNBC | Avelumab(10 mg/kg, IV) | standard surgery + (Neo)adj chemotherapy → Avelumab | 466 | ITT: 3-year DFS 68.3% vs 63.2% | complete |
| CABIN (ChiCTR2200067005) | Ⅱ | Early or locally advanced TNBC | Cadonilimab(10 mg/kg, IV) | Cadonilimab + nab-Paclitaxel + Carboplatin | 29 | tpCR: 65.5% | complete |
| cTRIO (ChiCTR2100041675) | Ⅱ | Early-stage TNBC | Tislelizumab(200 mg, IV, Day 1 of each cycle) | Tislelizumab + nab-Paclitaxel + Carboplatin → Tislelizumab | 62 | pCR: 56% | complete |
| MIRINAE (NCT03756298) | Ⅱ | Early-stage TNBC | Atezolizumab(1200 mg, IV) | Capecitabine ± Atezolizumab | 305 | Not reported yet | complete |

**Supplementary Table 1.** Clinical trials of ICIs targeting PD-1/PD-L1 in TNBC. This table summarizes the major clinical trials investigating PD-1/PD-L1 inhibitors in triple-negative breast cancer (TNBC), covering different disease settings, treatment regimens, sample sizes, key efficacy outcomes, and trial status. It highlights the distinct therapeutic activity of immune checkpoint inhibitors in early-stage and metastatic TNBC

| Trial | Phase | Setting | PD1/PD-L1 inhibitor | Drug administration regimen | RT mode | Sample size | Result | Synchronous OR Sequential | Status |
| --- | --- | --- | --- | --- | --- | --- | --- | --- | --- |
| TONIC (NCT02499367） | Ⅱ | Metastatic TNBC | Nivolumab (3 mg/kg, IV) | Radiotherapy / Doxorubicin / Cyclophosphamide / Cisplatin / No induction treatment + Nivolumab | SBRT 3 × 8 Gy | 67 | ORR:8% vs 35% vs 8% vs 23% vs 17% | Sequential | complete |
| NCT02730130 | II | Metastatic TNBC | Pembrolizumab (200 mg, IV) | Radiotherapy + Pembrolizumab | 5 × 600 cGy | 17 | ORR: 17.6% | Synchronous | complete |
| PEARL (NCT03366844) | Ib/II | Early stage TNBC | Pembrolizumab (200 mg, IV) | Radiotherapy + Pembrolizumab + NAC | SBRT 3 × 8 Gy | 54 | Incidence of grade ≥ 3 toxicities: 41%; TILs: 17.1% | Synchronous | complete |
| NCT05132790 | II | Early stage TNBC | Adebrelimab (20 mg/kg, IV) | Adebrelimab + Radiotherapy → Adebrelimab + nab-Paclitaxel + Carboplatin | SBRT 3 × 8 Gy | 10 | pCR: 90%; RCB 0-I: 100% | Synchronous | complete |
| NCT04683679 | II | Metastatic TNBC | Pembrolizumab (200 mg, IV) | Pembrolizumab + Radiotherapy ± Olaparib | SBRT 3 × 8-9 Gy | 34 | Not reported yet | Synchronous | Recruiting |
| NCT05491226 | II | High-risk TNBC | Pembrolizumab (200 mg, IV) | Pembrolizumab + Radiotherapy + Axatilimab | SBRT 3 × 8Gy | 35 | Not reported yet | Synchronous | Active, not recruiting |
| NCT03004183 | II | Metastatic TNBC | Pembrolizumab (200 mg, IV) | ADV/HSV-tk → Valacyclovir + Radiotherapy → Pembrolizumab | SBRT 5 × 6 Gy | 57 | Not reported yet | Sequential | complete |
| NCT04837209 | II | Metastatic TNBC | Dostarlimab (500 mg, IV, the first four cycles; 1000 mg, IV, follow-up | Niraparib + Dostarlimab + Radiotherapy | SBRT 3 × 8 Gy | 32 | Not reported yet | Synchronous | Active, not recruiting |
| BRINK (NCT07188246) | I/II | Early stage TNBC | Pembrolizumab (2 mg, Intratumor Injection) | Radiotherapy → Pembrolizumab → Surgery | SBRT (The dosage remains to be determined) | 40 | Not reported yet | Sequential | Recruiting |
| ASTRAEA (NCT07015853) | II | Metastatic TNBC | Pembrolizumab (200 mg, IV) | Pembrolizumab + Radiotherapy → Axatilimab | SBRT 3 × 8 Gy | 34 | Not reported yet | Synchronous | Not yet recruiting |
| NCT06735131 | II | Metastatic TNBC | Toripalimab (240 mg, IV) | Radiotherapy → Toripalimab + Nab-paclitaxel / Gemcitabine + Carboplatin | 5 × 5 Gy; 5 × 8 Gy; 3 × 8 Gy; 3 × 10 Gy; 0.5 Gy twice-a-day × 2 days, repeat for 4 cycles | 60 | Not reported yet | Sequential | Recruiting |
| NCT06627712 | Ⅲ | Early stage TNBC | Not reported yet | Not reported yet | SBRT 3 × 8Gy | 318 | Not reported yet | Not reported yet | Recruiting |
| NCT06238921 | I/II | Metastatic TNBC with brain metastases | Zimberelimab (360 mg, IV) | Radiotherapy → Zimberelimab + Sacituzumab govitecan | SRS (The dosage remains to be determined) | 31 | Not reported yet | Sequential | Recruiting |
| NCT03818685 | II | Early stage TNBC | Nivolumab (360 mg, IV); Ipilimumab (1 mg/kg, IV) | Radiotherapy + Nivolumab + Ipilimumab / Capecitabine | The dosage remains to be determined | 95 | Not reported yet | Not reported yet | Terminated |

Supplementary Table 2. Clinical trials of ICIs targeting PD-1/PD-L1 plus RT for TNBC. This table summarizes the current clinical studies evaluating the combination of radiotherapy and PD-1/PD-L1 inhibitors in TNBC, with emphasis on treatment setting, radiation mode, sequencing strategy, efficacy outcomes, and trial status. It reflects the ongoing exploration of the optimal therapeutic window for radioimmunotherapy.
